# Prevalence, Clinical Characteristics, and Braden Risk Categories of Pressure Injuries Among Patients Admitted to a Palliative Care Unit: A Retrospective Single-Center Study

**DOI:** 10.3390/healthcare14162449

**Published:** 2026-08-07

**Authors:** Nagihan Yıldız Çeltek, Tuğba Özdursun Karaca

**Affiliations:** 1 Department of Family Medicine, Faculty of Medicine, Tokat Gaziosmanpasa University, 60030 Tokat, Turkey; 2 Department of Palliative Care, Tokat Gaziosmanpasa University Hospital, 60030 Tokat, Turkey

**Keywords:** Braden Scale, palliative care, pressure injuries, clinical characteristics, retrospective study

## Abstract

**Highlights:**

**What are the main findings?**

- The prevalence of documented pressure injuries among patients admitted to the palliative care unit was 14.36%.
- Among patients with documented pressure injuries, 77.8% had pressure injuries at admission, whereas 22.2% developed pressure injuries during hospitalization.
- Several clinical and care-related characteristics were associated with Braden risk categories. Primary diagnosis, caregiver relationship, level of consciousness, and mobility status were associated with whether pressure injuries were present at admission or developed during hospitalization.
- In multivariable analysis, longer length of stay and care by a second-degree relative were associated with pressure injury development during hospitalization.

**What are the implications of the main findings?**

- Regular Braden-based risk assessment and systematic skin evaluation should be conducted in palliative care units.
- Individualized preventive interventions should be implemented according to patients’ mobility, nutritional status, elimination pattern, consciousness level, and medical device use.
- Nurses and healthcare team members working in palliative care units should receive regular in-service training on pressure injury prevention, risk assessment, and care.

**Abstract:**

Background/Objectives: Pressure injuries remain a significant clinical problem in palliative care. Understanding their prevalence and associated clinical characteristics can support early recognition and prevention strategies. This study aimed to determine the prevalence of pressure injuries among patients admitted to a palliative care unit and to examine the clinical and care-related characteristics associated with Braden risk categories and pressure injury status at admission or during hospitalization. Methods: This retrospective, descriptive, and analytical study was conducted between 1 April 2018, and 30 November 2023. Among 1942 patients hospitalized in the Palliative Care Unit of Tokat Gaziosmanpaşa University Hospital, the medical records of 279 patients with documented pressure injuries were retrospectively reviewed. The collected data included patients’ demographic characteristics, clinical and care-related characteristics, pressure injury status, and Braden Pressure Injury Risk Assessment Scale scores. Bivariate analyses and multivariable binary logistic regression analysis were performed. Results: The prevalence of pressure injuries was 14.36%. Of the patients with pressure injuries, 217 (77.8%) had pressure injuries at the time of admission to the unit, while 62 (22.2%) developed pressure injuries during hospitalization. Age, use of medical devices and invasive procedures, nutritional status, urinary and fecal elimination status, comorbidity status, mobility status, level of consciousness, and primary diagnosis were significantly associated with Braden risk categories. Primary diagnosis, caregiver relationship, level of consciousness, and mobility status were significantly associated with whether pressure injuries were present at admission or developed during hospitalization. In multivariable analysis, longer length of stay and care by a second-degree relative were associated with pressure injury development during hospitalization, whereas acute trauma diagnosis and immobility were associated with pressure injuries already being present at admission. Conclusions: The findings indicate that several clinical and care-related characteristics were associated with higher Braden risk categories and with whether pressure injuries were present at admission or developed during hospitalization. Because the study included only patients with documented pressure injuries, the results should be interpreted as associations within this patient group rather than as evidence of independent risk factors for pressure injury development.

## 1. Introduction

According to the World Health Organization (WHO), palliative care is defined as “an approach that improves the quality of life of patients and their relatives facing problems associated with life-threatening illness, through the prevention and relief of suffering by means of early identification, impeccable assessment, and treatment of pain and other problems, including physical, psychosocial, and spiritual needs” [1,2].

Globally, palliative care services are delivered through various organizational models. Although substantial progress has been made in developed countries, palliative care systems in many developing countries remain in the process of expansion and integration. Türkiye is classified as a Group 3b country, indicating that palliative care services are available at the local or regional level. In this category, financial support is provided through a combination of personal donations, public and private health insurance systems, and local governmental resources, while professional associations and organizations contribute primarily by supporting educational activities [1,3].

The primary objective of palliative care is to optimize quality of life by relieving physical symptoms, particularly pain and pressure injuries, through both pharmacological and non-pharmacological interventions, while also addressing patients’ psychological, social, and economic needs. Among these symptoms, pressure injuries represent a major clinical challenge because they prolong hospitalization, increase treatment burden, and contribute to higher morbidity and mortality, ultimately compromising patients’ quality of life and comfort. Consequently, the prevention of pressure injuries is a fundamental priority in palliative care practice [4,5].

Early identification of patients at risk is essential for preventing pressure injuries and minimizing tissue damage. The guidelines developed by the European Pressure Ulcer Advisory Panel (EPUAP), the National Pressure Ulcer Advisory Panel (NPUAP), and the Pan Pacific Pressure Injury Alliance (PPPIA) recommend that pressure injury risk should be assessed using reliable and validated patient-specific risk assessment tools in combination with comprehensive clinical and skin evaluations [4,6,7].

Several risk assessment tools are currently used in clinical practice, including the Braden, Norton, and Waterlow Pressure Injury Risk Assessment Scales. Comparative studies have demonstrated variability in their diagnostic performance. Balzer et al. reported that the Waterlow Scale had the highest sensitivity (0.86), whereas the Norton Scale demonstrated the highest specificity (0.75) [8]. Similarly, Kwong et al. compared the revised Braden Scale with the Norton Scale in a Chinese population and found that the Braden Scale achieved superior sensitivity (89%) and specificity (75%) [9,10].

A comprehensive understanding of the factors associated with pressure injury development is essential for effective prevention and management. The principal causative factor is prolonged pressure on tissue, followed by reduced tissue tolerance to mechanical loading [11,12]. Risk factors are generally classified as extrinsic and intrinsic. Extrinsic factors include pressure, shear, friction, and moisture, all of which directly contribute to tissue damage [4,6,7]. Intrinsic factors comprise patient-related characteristics such as advanced age, immobility, impaired consciousness, malnutrition, chronic diseases, and sensory impairment, all of which increase susceptibility to pressure injury development [3,4].

Despite appropriate preventive interventions, pressure injuries cannot always be avoided, particularly in patients with advanced illness. Accurate wound assessment and staging are therefore essential for selecting appropriate treatment strategies and guiding clinical management. Pressure injury staging provides information on the extent of tissue damage, wound depth, and the involvement of underlying structures, including subcutaneous tissue, muscle, and bone [13,14,15].

In palliative care settings, pressure injuries should be considered within the broader context of advanced disease, progressive functional decline, and end-of-life care. Patients admitted to palliative care units commonly present with multiple comorbidities, poor nutritional intake, limited mobility, impaired consciousness, incontinence, edema, and frequent use of medical devices. The coexistence of these factors substantially increases the complexity of both prevention and management. Accordingly, effective pressure injury care in palliative settings extends beyond wound treatment and requires comprehensive risk assessment, symptom control, skin protection, nutritional support, moisture management, regular repositioning, and individualized care planning [16,17].

Pressure injuries are classified according to the severity and extent of tissue damage. Stage I is characterized by intact skin with localized non-blanchable erythema. Stage II involves partial-thickness skin loss with exposure of the dermis and typically presents as a superficial ulcer. Stage III is defined by full-thickness skin loss extending into the subcutaneous tissue, with visible adipose tissue and granulation tissue. Stage IV represents full-thickness tissue loss involving deeper anatomical structures, including muscle, tendon, ligament, cartilage, or bone. Unstageable pressure injury refers to full-thickness tissue loss in which the wound bed is completely obscured by slough or eschar, preventing accurate assessment of wound depth; after removal of necrotic tissue, these injuries are generally classified as Stage III or Stage IV. Deep tissue pressure injury is characterized by persistent non-blanchable deep red, maroon, or purple discoloration, or epidermal separation revealing a dark wound bed or blood-filled blister. Mucosal membrane pressure injury occurs on mucous membranes at sites exposed to medical devices [6,7].

The present study aimed to determine the prevalence of pressure injuries among patients admitted to a palliative care unit and to evaluate the clinical and care-related factors associated with Braden risk categories and pressure injury status at admission or during hospitalization. We hypothesized that Braden risk categories and pressure injury status differ according to patient characteristics, including age, primary diagnosis, comorbidity burden, nutritional status, elimination pattern, level of consciousness, mobility status, and the use of medical devices.

## 2. Materials and Methods

### 2.1. Study Population and Protocol

This retrospective descriptive study was conducted in the Palliative Care Unit of Tokat Gaziosmanpaşa University Hospital. Between 1 April 2018, and 30 November 2023, a total of 1942 patients were admitted to the unit and screened for eligibility. Patients were included if they had a documented pressure injury recorded in the hospital information system, the pressure injury had been reported to the hospital quality control unit, and complete records were available for all study variables.

A documented pressure injury was defined as an injury recorded in the hospital information system, reported to the hospital quality control unit, and supported by corresponding documentation in the medical or nursing records and the Braden Pressure Injury Assessment and Follow-up Form. Information on pressure injury status, anatomical location, stage, and the date of first documentation was extracted from these records. Pressure injuries documented during the initial admission assessment were classified as present at admission, whereas those first documented after admission were classified as developed during hospitalization.

Among the 1942 patients screened, 279 met the inclusion criteria and had a documented pressure injury, whereas 1663 patients had no documented pressure injury and were therefore not included in the analytical sample. No additional patients were excluded because of missing or inaccessible data. The final study population consisted of 128 women and 151 men. Of the included patients, 217 had a pressure injury documented at admission, whereas 62 developed a pressure injury during hospitalization.

Demographic, clinical, and care-related data were retrospectively collected from the hospital information system, medical and nursing records, and the routinely completed Braden Pressure Injury Assessment and Follow-up Form. The collected variables included age, sex, body mass index, marital status, place of residence, caregiver relationship, primary diagnosis, comorbidity status, nutritional status, urinary and fecal elimination status, level of consciousness, edema, mobility status, medical device use, pressure injury location and stage, and Braden Scale score.

The caregiver relationship was determined from the caregiver information recorded in the patient file at admission. In the study dataset, this variable was coded as 1 = first-degree relative, 2 = second-degree relative, and 3 = care worker. These categories were analyzed according to their original coding in the patient records.

### 2.2. Ethical Considerations

Ethical approval for this study was obtained from the Clinical Research Ethics Committee of Tokat Gaziosmanpaşa University (Approval No. 23-KAEK-292; Decision No. 83116987-781; 21 December 2023). Institutional permission was also obtained from Tokat Gaziosmanpaşa University Hospital (Document No. E-21979232-020-382092; 8 January 2024).

### 2.3. Statistical Analysis

Descriptive statistics were used to summarize the general characteristics of the study population. Continuous variables are presented as mean ± standard deviation (SD), whereas categorical variables are expressed as frequency (*n*) and percentage (%).

Data completeness was assessed before statistical analysis. All variables included in the final analyses were complete for the 279 patients; therefore, no missing-data imputation was required. Continuous variables, including age, height, body weight, body mass index, and length of hospital stay, were analyzed using complete-case data.

Comparisons of continuous variables between two groups were performed using the Independent Samples *t*-test, whereas comparisons involving more than two groups were conducted using one-way analysis of variance (ANOVA). Associations between categorical variables were evaluated using cross-tabulations and the chi-square test.

In addition to the bivariate analyses, a multivariable binary logistic regression analysis was performed to identify clinical and care-related characteristics associated with pressure injury status among patients with documented pressure injuries. The dependent variable was coded as 0 = pressure injury present at admission and 1 = pressure injury developed during hospitalization. Because the study population included only patients with documented pressure injuries, it was not possible to construct a model comparing patients with and without pressure injuries.

Primary diagnosis, caregiver relationship, level of consciousness, length of hospital stay, and mobility status were included in the final model based on their clinical relevance and the findings of the bivariate analyses. All variables were entered simultaneously using the Enter method, and no automated forward or backward stepwise selection procedure was applied.

Multicollinearity was assessed using variance inflation factor (VIF) values. Overall model significance was evaluated using the omnibus likelihood-ratio test, model explanatory performance was assessed using the Cox–Snell and Nagelkerke R^2^ statistics, goodness of fit was evaluated with the Hosmer–Lemeshow test, and model discrimination was assessed by the area under the receiver operating characteristic (ROC) curve. Results are presented as adjusted odds ratios (aORs), 95% confidence intervals (CIs), and *p*-values. A two-sided *p*-value < 0.05 was considered statistically significant. All statistical analyses were performed using IBM SPSS Statistics for Windows, version 22.0 (IBM Corp., Armonk, NY, USA).

## 3. Results

A total of 279 patients were included in the study. The mean age was 72.35 ± 12.90 years (range, 27–98 years), and 45.9% were female. Among caregivers, 86.4% were first-degree relatives. Regarding primary diagnoses, 62.7% of the patients had an oncological disease. More than half of the patients with pressure injuries (52.2%) were admitted from home. The mean length of stay in the palliative care unit was 23.18 ± 19.86 days (range, 2–145 days). The mean height was 166.42 ± 8.73 cm (range, 150–188 cm), the mean body weight was 59.88 ± 13.11 kg (range, 38–130 kg), and the mean body mass index (BMI) was 21.63 ± 4.81 kg/m^2^ (range, 14.61–54.11 kg/m^2^). BMI did not differ significantly between patients with pressure injuries present at admission and those who developed pressure injuries during hospitalization (21.89 ± 5.02 vs. 20.75 ± 3.89 kg/m^2^, respectively; t = 1.644, *p* = 0.101).

Clinical and care-related characteristics potentially associated with Braden risk categories and pressure injury status were evaluated in all 279 patients with documented pressure injuries. Comorbidities were present in 61.3% of the study population, with 20.8% having cardiovascular diseases and 19.0% having both internal medicine and cardiovascular conditions. Regarding nutritional status, 41.6% of the patients received oral nutrition, whereas 20.4% were unable to tolerate oral intake and were fed exclusively via the parenteral route. Urinary elimination was managed with a Foley catheter in 82.1% of patients, spontaneous voiding in 8.2%, absorbent pads in 7.5%, and urinary ostomies (e.g., nephrostomy or cystofix) in 2.2%. Fecal elimination occurred through absorbent pads in 75.6% of patients, spontaneous defecation in 19.7%, and intestinal ostomies (e.g., colostomy or ileostomy) in 4.7%.

Regarding the level of consciousness, 39.8% of patients were alert, 43.4% were confused, and 16.8% were unconscious. Only 0.7% were fully mobile, whereas 17.6% were able to stand with assistance, 18.6% were able to turn partially in bed, and 63.1% were completely immobile. Edema was present in 55.2% of patients, including generalized edema in 28.3% and lower-extremity edema in 21.5%.

The distribution of invasive procedures and medical devices that could potentially contribute to pressure injury risk was also evaluated. All patients (100%) had intravenous access, and 87.1% had a Foley catheter in place. Medical devices located in the facial or cervical region, including nasogastric tubes, nebulizer masks, oxygen masks, and nasal cannulas, were present in 58.8% of patients. In addition, 18.6% had abdominal or dorsal ostomies or drainage systems that could increase pressure injury risk, while 5.7% had a tracheostomy.

Overall, 62 patients (22.2%) developed pressure injuries during hospitalization, whereas 217 patients were admitted with pre-existing pressure injuries. Because some patients had pressure injuries at more than one anatomical site, each location was analyzed separately. The sacral region was the most frequently affected site, occurring in 84.9% of patients. Pressure injuries were also identified at the heel (19.0%), trochanter (18.6%), scapula (11.1%), foot and toes (3.6%), periauricular region (2.9%), elbow (1.8%), patella (1.8%), and other anatomical locations (3.9%).

The distribution of Braden Pressure Injury Risk Scale (BPIRS) categories according to selected clinical and care-related characteristics is presented in Table 1. BPIRS categories were significantly associated with the presence of comorbidities (χ^2^ = 11.557, *p* = 0.009). Patients with comorbidities were more likely to be classified as having a high BPIRS than those without comorbidities (69.0% vs. 53.7%, *p* < 0.05).

BPIRS categories also differed significantly according to nutritional status (χ^2^ = 34.957, *p* < 0.001). Patients receiving jejunostomy feeding had the highest proportion of high BPIRS scores (94.1%), which was significantly greater than that observed among patients receiving oral nutrition (49.1%), parenteral nutrition (64.9%), or combined oral and parenteral nutrition (51.6%) (*p* < 0.05). Likewise, patients fed via a nasogastric tube had a significantly higher proportion of high BPIRS scores (82.9%) than those receiving oral nutrition (49.1%) or combined oral and parenteral nutrition (51.6%) (*p* < 0.05).

A significant association was observed between BPIRS categories and the method of urinary elimination (χ^2^ = 23.784, *p* = 0.005). Patients managed with a Foley catheter were more frequently classified as having a high BPIRS than those with spontaneous urination (67.2%).

BPIRS categories were also significantly associated with fecal elimination status (χ^2^ = 34.902, *p* < 0.001). The proportion of patients with a high BPIRS was significantly greater among those using absorbent pads for fecal elimination (71.6%) than among those with spontaneous defecation (34.5%) (*p* < 0.05).

A significant relationship was found between BPIRS categories and level of consciousness (χ^2^ = 71.948, *p* < 0.001). Patients who were unconscious had the highest proportion of high BPIRS scores (93.6%), followed by confused patients (77.7%) and conscious patients (34.2%) (*p* < 0.05).

No significant association was observed between BPIRS categories and the presence of edema (χ^2^ = 5.693, *p* = 0.128).

Mobility status was significantly associated with BPIRS categories (χ^2^ = 54.235, *p* < 0.001). The proportion of patients with high BPIRS scores was significantly greater among immobile patients (76.1%) than among those who were able to turn in bed (51.9%) or ambulate with assistance (30.6%) (*p* < 0.05).

The distribution of Braden risk categories according to primary diagnosis and admission source is presented in Table 2. Braden risk categories differed significantly according to primary diagnosis (χ^2^ = 41.291, *p* < 0.001). Among the diagnostic groups, patients with neurological diseases (87.9%) and those with acute trauma (86.7%) had significantly higher proportions of high Braden risk than patients with oncological diseases (50.3%) (*p* < 0.05).

Admission source was also significantly associated with Braden risk categories (χ^2^ = 22.518, *p* = 0.032). Patients admitted from the intensive care unit were more frequently classified as having a high Braden risk than those transferred from another hospital ward (84.6% vs. 52.9%, *p* < 0.05).

Patient age differed significantly across Braden risk categories (F = 3.834, *p* = 0.010). Patients classified as having a high Braden risk were significantly older than those in the low-risk category (Table 3).

Foley catheter use was significantly associated with BPIRS categories (χ^2^ = 15.096, *p* = 0.002). Patients with a Foley catheter were more frequently classified as having a high BPIRS than those without a Foley catheter (66.7% vs. 38.9%, *p* < 0.05). BPIRS categories were also significantly associated with the use of a nasogastric tube, nasal cannula, or oxygen mask (χ^2^ = 8.471, *p* = 0.037). Patients using at least one of these devices had a significantly higher proportion of high BPIRS scores than those without these devices (70.1% vs. 53.0%, *p* < 0.05) (Table 4).

There is no statistically significant association between BPIRS and the pressure injury status (χ^2^ = 5.428; *p* = 0.143) (Table 5).

Primary diagnosis was significantly associated with the stage distribution of sacral pressure injuries (χ^2^ = 64.065, *p* < 0.001). Patients with acute trauma had significantly higher proportions of Stage 4 (13.3% vs. 1.7%) and Stage 5 (16.7% vs. 1.1%) sacral pressure injuries than patients with oncological diseases (*p* < 0.05 for both comparisons) (Table 6).

Primary diagnosis was also significantly associated with the stage distribution of trochanteric pressure injuries (χ^2^ = 47.443, *p* = 0.001). The proportion of Stage 3 trochanteric pressure injuries was significantly higher among patients with acute trauma (10.0%) and those with cardiovascular diseases (20.0%) than among patients with oncological diseases (0.6%) (*p* < 0.05).

A significant association was likewise observed between primary diagnosis and the stage distribution of heel pressure injuries (χ^2^ = 54.886, *p* < 0.001). Patients with acute trauma had a significantly higher proportion of Stage 2 heel pressure injuries than patients with oncological diseases (16.7% vs. 5.1%, *p* < 0.05) (Table 6).

Pressure injury status at admission was significantly associated with patients’ primary diagnosis (χ^2^ = 18.397, *p* = 0.001). Patients with acute trauma were more likely to have a pressure injury present at admission than patients with oncological diseases (93.3% vs. 69.7%, *p* < 0.05). Conversely, among patients who developed a pressure injury during hospitalization, the proportion of those with oncological diseases was significantly higher than that of patients with acute trauma (30.3% vs. 6.7%, *p* < 0.05) (Table 7).

Caregiver relationship was significantly associated with pressure injury development during hospitalization (χ^2^ = 7.249, *p* = 0.027). Among patients cared for by second-degree relatives, the proportion who developed a pressure injury during hospitalization was significantly higher than the proportion admitted with a pre-existing pressure injury (16.1% vs. 6.5%, *p* < 0.05) (Table 8).

Level of consciousness was significantly associated with pressure injury status at admission (χ^2^ = 8.972, *p* = 0.011). Patients who were unconscious were more likely to have a pressure injury present at admission than those who were confused or conscious (93.6% vs. 76.9% and 72.1%, respectively; *p* < 0.05) (Table 9).

Mobility status was significantly associated with pressure injury status at admission (χ^2^ = 20.217, *p* < 0.001). Patients who were immobile were more likely to have a pressure injury present at admission than those who were able to turn in bed or ambulate with assistance (85.8% vs. 67.3% and 59.2%, respectively; *p* < 0.05) (Table 10).

The overall multivariable model was statistically significant compared with the intercept-only model (likelihood-ratio χ^2^(9) = 45.538, *p* < 0.001). The Cox–Snell and Nagelkerke R^2^ values were 0.151 and 0.230, respectively. Model discrimination was acceptable, with an area under the receiver operating characteristic curve of 0.787 (95% CI: 0.724–0.844). No evidence of substantial multicollinearity was observed, as all variance inflation factor values were below 4. However, the Hosmer–Lemeshow goodness-of-fit test was statistically significant (χ^2^(8) = 18.657, *p* = 0.017), indicating some discrepancy between the observed and predicted probabilities. Accordingly, the adjusted odds ratios should be interpreted with caution.

A multivariable binary logistic regression analysis was performed to identify clinical and care-related factors associated with pressure injury status among patients with documented pressure injuries. The dependent variable was pressure injury status, coded as 0 = pressure injury present at admission and 1 = pressure injury developed during hospitalization. In the adjusted model, patients with acute trauma had lower odds of developing a pressure injury during hospitalization than those with oncological diseases (adjusted OR = 0.21; 95% CI: 0.07–0.67; *p* = 0.008). Patients cared for by second-degree relatives had higher odds of developing a pressure injury during hospitalization than those cared for by first-degree relatives (adjusted OR = 2.93; 95% CI: 1.12–7.71; *p* = 0.029). Longer hospital stay was also associated with increased odds of pressure injury development during hospitalization (adjusted OR = 1.02; 95% CI: 1.01–1.04; *p* = 0.005). Likewise, immobility was associated with lower odds of pressure injury development during hospitalization (adjusted OR = 0.44; 95% CI: 0.21–0.91; *p* = 0.027). However, this finding should be interpreted cautiously because immobile patients were more likely to have pressure injuries already present at admission. Therefore, these findings should be interpreted as factors associated with pressure injury status among patients with documented pressure injuries rather than as predictors of pressure injury occurrence in the overall palliative care population (Table 11).

## 4. Discussion

This retrospective study investigated the prevalence of documented pressure injuries and examined the clinical and care-related characteristics associated with Braden risk categories and pressure injury status at admission or during hospitalization among patients admitted to a palliative care unit. Sociodemographic characteristics, clinical features, and findings from the Braden Pressure Injury Risk Assessment Form were evaluated to explore factors associated with pressure injury risk and status. The findings were interpreted in the context of the existing literature and current evidence.

Among the 1942 patients admitted to the palliative care unit during the study period, 279 (14.36%) had documented pressure injuries. Of these, 217 patients presented with pressure injuries at admission, whereas 62 (22.2%) developed pressure injuries during hospitalization. Previous studies conducted in intensive care units and hospital wards in Türkiye have reported pressure injury rates ranging from 18.3% to 28.6% [18,19,20]. Although direct comparisons should be made cautiously because of differences in study populations and clinical settings, the prevalence observed in our study falls within the range reported for hospitalized patients in Türkiye. However, because studies specifically evaluating pressure injury prevalence in palliative care settings in Türkiye remain limited, direct comparisons with similar patient populations are currently not possible.

The present study also examined the associations between patients’ demographic and clinical characteristics, Braden risk categories, and pressure injury status at admission and during hospitalization.

Age

Advanced age is a well-established risk factor for pressure injury development. Previous studies have shown that approximately 70% of patients older than 70 years develop pressure injuries within two weeks of hospital admission [5]. Similarly, another study reported that the risk increases after the age of 51 and becomes substantially higher among patients older than 65 years [21]. More than half of the patients with pressure injuries were reported to be over 70 years of age, a finding attributed to age-related physiological changes, including thinning of the epidermis and reduced dermal vascularization [5,11]. Another study found that although pressure injuries occurred in 20.56% of the overall study population, the prevalence increased to 31.4% among patients aged 75 years or older [5,22]. Collectively, these findings support the role of advanced age as an important risk factor for pressure injury development.

Consistent with previous reports, patients classified as having a high Braden risk in the present study were older than those in the lower-risk categories. This finding suggests that advanced age is associated with greater pressure injury risk among palliative care patients with documented pressure injuries.

Gender

Previous studies have reported inconsistent findings regarding the relationship between sex and pressure injury development. Efteli and Yapucu reported pressure injuries in 20% of female patients and 36% of male patients [19], whereas Kurtuluş found a higher frequency of pressure injuries among men aged 65 years and older, although the difference did not reach statistical significance [23]. In the present study, sex was not associated with either Braden risk categories or pressure injury status. The proportions of female and male patients with documented pressure injuries were similar, suggesting that sex was not a clinically relevant distinguishing factor in this study population.

The present study further explored the relationships between pressure injury status and several clinical characteristics, including primary diagnosis, comorbidity status, nutritional status, body mass index, elimination pattern, mobility status, and level of consciousness.

Diagnosis

Previous studies have examined pressure injury risk across different diagnostic groups. A study conducted in 2019 reported that pressure injuries occurred most frequently among patients with respiratory diseases (35.9%), followed by those with oncological diseases (25.0%), acute renal failure (14.3%), cirrhosis (14.3%), and cardiovascular diseases (13.6%) [24]. In the present study, primary diagnosis was significantly associated with Braden risk categories. Patients with neurological diseases and acute trauma were more frequently classified as having a high Braden risk than those with oncological diseases. In addition, sacral pressure injuries were more common among patients with acute trauma, whereas Stage 3 trochanteric pressure injuries occurred more frequently in patients with acute trauma and cardiovascular diseases. These findings likely reflect differences in functional status, mobility limitations, and the underlying clinical characteristics of these patient groups.

Comorbidity Status

Comorbid conditions, particularly cardiovascular and peripheral vascular diseases, have consistently been identified as important contributors to pressure injury development [15,25,26]. Impaired tissue perfusion, reduced mobility, and increased care dependency associated with these conditions may further increase susceptibility to pressure injury.

Consistent with previous reports, more than half of the patients in our study had at least one comorbid condition. Patients with comorbidities were more frequently classified as having a high Braden risk than those without comorbidities, suggesting that the cumulative burden of chronic disease contributes to an increased risk profile among palliative care patients.

Nutrition and Body Mass Index

Nutritional status plays a central role in maintaining skin integrity and supporting wound healing. Malnutrition contributes to pressure injury development through muscle wasting, loss of subcutaneous tissue, impaired collagen synthesis, and reduced skin elasticity, whereas obesity may increase pressure over bony prominences and exacerbate friction and shear forces [12,13,27].

Although previous studies have identified body mass index as a potential risk factor for pressure injury development, BMI was not associated with pressure injury status at admission or during hospitalization in the present study. This finding suggests that, in palliative care patients, nutritional status and overall clinical condition may have a greater influence on pressure injury risk than BMI alone.

Elimination Pattern

Urinary and fecal incontinence are well-recognized risk factors for pressure injury development because prolonged exposure to moisture and chemical irritation compromises skin integrity [28,29]. Patients with fecal incontinence are particularly vulnerable because persistent skin moisture promotes maceration and increases susceptibility to tissue damage.

In the present study, Foley catheter use and fecal elimination managed with absorbent pads were both associated with higher Braden risk categories. These findings likely reflect greater functional dependence and impaired continence among patients with documented pressure injuries rather than indicating independent causal risk factors.

Mobility Status

Immobility is one of the most important determinants of pressure injury development and is frequently associated with advanced age, multiple comorbidities, and functional decline [2,30,31]. Reduced mobility prolongs pressure over bony prominences and limits spontaneous repositioning, thereby increasing the likelihood of tissue injury.

Consistent with previous studies, most patients with documented pressure injuries in our cohort were immobile, and immobility was strongly associated with high Braden risk categories. Multivariable analysis further demonstrated that longer hospital stay and care provided by a second-degree relative were independently associated with pressure injury development during hospitalization. In contrast, acute trauma and immobility were associated with lower odds of pressure injury development during hospitalization. This finding most likely reflects the fact that these patients were more frequently admitted with pre-existing pressure injuries rather than developing new injuries after admission. Therefore, these associations should not be interpreted as protective effects but rather as differences in pressure injury status at admission versus during hospitalization.

Level of Consciousness

Impaired consciousness and sensory deficits are well-established risk factors for pressure injury development because affected patients are often unable to recognize discomfort or reposition themselves in response to prolonged pressure [32,33]. This risk is particularly pronounced in patients with neurological impairment, including paraplegia and quadriplegia, who may have substantial sensory loss and reduced spontaneous mobility.

Mollaoğlu et al. reported that more than half of the patients hospitalized with cerebral vascular hemorrhage were classified as being at very high risk for pressure injury [33]. Similarly, in the present study, unconscious patients were more frequently classified as having a high Braden risk than conscious or confused patients.

However, these findings should be interpreted with caution. Several variables evaluated in this study—including mobility status, nutritional route, level of consciousness, and elimination-related characteristics—correspond directly or conceptually to components of the Braden Scale, including the mobility, activity, nutrition, sensory perception, and moisture domains. Consequently, their associations with overall Braden risk categories are expected and do not represent newly identified or independent clinical risk factors. Rather, these findings describe the clinical characteristics that contribute to the calculation of the Braden risk score.

Limitations

This study has several limitations that should be considered when interpreting the findings. First, it was a retrospective, single-center study conducted in a palliative care unit, which may limit the generalizability of the results to other palliative care settings. Second, only patients with documented pressure injuries were included, and no comparison group of patients without pressure injuries was available. Consequently, the findings should be interpreted as associations observed among patients with documented pressure injuries rather than as evidence of independent risk factors for pressure injury development.

Furthermore, several variables examined in relation to the overall Braden risk category—including mobility status, nutritional route, level of consciousness, and elimination status—correspond directly or conceptually to domains of the Braden Scale. Therefore, the observed associations are partly inherent to the structure of the scale and should not be interpreted as independent risk-factor effects.

Another limitation is that pressure injuries were identified from documentation in the hospital information system and reports submitted to the hospital quality control unit. As a result, underreporting and ascertainment bias cannot be excluded. Finally, several clinically relevant variables, including the duration of immobility, severity of the underlying disease, laboratory markers reflecting nutritional and inflammatory status, and detailed information on preventive care interventions, were not consistently available in the retrospective records and therefore could not be incorporated into the analyses.

## 5. Conclusions

This retrospective single-center study found that the prevalence of documented pressure injuries among patients admitted to a palliative care unit was 14.36%, with most injuries already present at admission. Primary diagnosis, caregiver relationship, level of consciousness, and mobility status were associated with pressure injury status and Braden risk categories. These findings highlight the importance of routine Braden-based risk assessment, comprehensive skin evaluation, and individualized preventive strategies in palliative care. Future prospective multicenter studies are needed to validate these findings and identify independent predictors of pressure injury development.

## Figures and Tables

**Table 1:** Distribution of Braden Risk Categories According to Clinical and Care-Related Characteristics among Patients with Documented Pressure Injuries.

Variables		Braden Risk Category								χ^2^-Value	*p*-Value
		12 and Below, High Risk		13–14, Moderate Risk		15–16, Low Risk		17 and Above, No Risk			
		*n*	%	*n*	%	*n*	%	*n*	%		
Comorbidity status	Yes	118	69.0 (a)	34	19.9	13	7.6	6	3.5 (a)	11.557	0.009
	No	58	53.7 (b)	23	21.3	14	13.0	13	12.0 (b)		
Comorbid Diseases	No comorbid disease	58	53.7	23	21.3	14	13.0	13	12.0	21.970	0.109
	Internal medicine disease	29	78.4	7	18.9	1	2.7	0	0.0		
	Cardiological disease	40	69.0	9	15.5	6	10.3	3	5.2		
	Neurological disease	9	69.2	1	7.7	2	15.4	1	7.7		
	Internal Medicine + Cardiology	36	67.9	13	24.5	3	5.7	1	1.9		
	Other	4	40.0	4	40.0	1	10.0	1	10.0		
Nutritional Status	Oral	57	49.1 (a)	33	28.4 (a)	16	13.8	10	8.6	34.957	<0.001
	Nasogastric	34	82.9 (bc)	5	12.2 (ab)	1	2.4	1	2.4		
	Gastrostomy, jejunostomy	32	94.1 (c)	0	0.0 (b)	2	5.9	0	0.0		
	Parenteral	37	64.9 (ab)	11	19.3 (ab)	5	8.8	4	7.0		
	Oral + parenteral	16	51.6 (a)	8	25.8 (a)	3	9.7	4	12.9		
Urinary Output Status	Normal	6	26.1 (a)	7	30.4	7	30.4 (a)	3	13.0	23.784	0.005
	Absorbent pad	12	57.1 (ab)	6	28.6	3	14.3 (ab)	0	0.0		
	Foley catheter	154	67.2 (b)	43	18.8	16	7.0 (b)	16	7.0		
	Cystofix/ostomy	4	66.7 (ab)	1	16.7	1	16.7 (ab)	0	0.0		
Stool Output Status	Normal	19	34.5 (a)	15	27.3	12	21.8 (a)	9	16.4 (a)	34.902	<0.001
	Absorbent pad	151	71.6 (b)	39	18.5	13	6.2 (b)	8	3.8 (b)		
	Cystofix/ostomy	6	46.2 (ab)	3	23.1	2	15.4 (ab)	2	15.4 (ab)		
State of Consciousness	Conscious	38	34.2 (a)	37	33.3 (a)	22	19.8 (a)	14	12.6 (a)	71.948	<0.001
	Confused	94	77.7 (b)	18	14.9 (b)	5	4.1 (b)	4	3.3 (b)		
	Unconscious	44	93.6 (c)	2	4.3 (b)	0	0.0 (b)	1	2.1 (ab)		
Presence of Edema	Yes	101	66.0	29	19.0	10	6.5	13	8.5	5.693	0.128
	No	75	59.5	28	22.2	17	13.5	6	4.8		
Edema location	Absent	74	59.2	28	22.4	17	13.6	6	4.8	21.651	0.117
	Lower extremity	34	56.7	16	26.7	5	8.3	5	8.3		
	Whole body	59	74.7	12	15.2	3	3.8	5	6.3		
	Upper extremity	6	60.0	0	0.0	2	20.0	2	20.0		
	Head neck	2	100.0	0	0.0	0	0.0	0	0.0		
	Other	1	33.3	1	33.3	0	0.0	1	33.3		
Mobility status	Mobile	0	0.0	1	50.0	1	50.0 (a)	0	0.0	54.235	<0.001
	Mobile with assistance	15	30.6 (b)	13	26.5	11	22.4 (a)	10	20.4 (b)		
	Mobile in bed	27	51.9 (b)	15	28.8	7	13.5 (ab)	3	5.8 (ab)		
	Immobile	134	76.1 (a)	28	15.9	8	4.5 (b)	6	3.4 (a)		

Pearson’s chi-square test was used. Letters in parentheses indicate pairwise comparisons between categories; values sharing the same letter are not significantly different. Statistical significance was set at *p* < 0.05.

**Table 2:** Distribution of Braden Risk Categories According to Primary Diagnosis and Admission Source.

Variables		Braden Risk Category								χ^2^-Value	*p*-Value
		12 and Upper, High Risk		13–14, Moderate Risk		15–16, Low Risk		17 and Above, No Risk			
		*n*	%	*n*	%	*n*	%	*n*	%		
Patient’s Diagnosis	Oncological	88	50.3 (a)	44	25.1	26	14.9	17	9.7	41.291	<0.001
	Acute trauma	52	86.7 (b)	6	10.0	1	1.7	1	1.7		
	Neurological	29	87.9 (b)	4	12.1	0	0.0	0	0.0		
	Cardiology	3	60.0 (ab)	2	40.0	0	0.0	0	0.0		
	Internal medicine	4	66.7 (ab)	1	16.7	0	0.0	1	16.7		
	Other	0	0.0	0	0.0	0	0.0	0	0.0		
Location where the patient was admitted to the ward	From home	91	62.3 (ab)	29	19.9	18	12.3 (ab)	8	5.5	22.518	0.032
	From nursing home	6	75.0 (ab)	0	0.0	2	25.0 (b)	0	0.0		
	From another ward	36	52.9 (b)	17	25.0	6	8.8 (ab)	9	13.2		
	From intensive care	33	84.6 (a)	5	12.8	0	0.0	1	2.6		
	From another center	10	55.6 (ab)	6	33.3	1	5.6 (ab)	1	5.6		

Pearson’s chi-square test was used. Letters in parentheses indicate pairwise comparisons between categories; values sharing the same letter are not significantly different. Statistical significance was set at *p* < 0.05.

**Table 3:** Age Distribution According to Braden Risk Categories.

	Braden Risk Category				F-Value	*p*-Value
	12 and Below, High Risk	13–14, Moderate Risk	15–16, Low Risk	17 and Upper, No Risk		
	Mean ± SD	Mean ± SD	Mean ± SD	Mean ± SD		
Age	73.78 ± 12.28 (a)	72.53 ± 12.74 (ab)	66.74 ± 15.85 (b)	66.53 ± 11.55 (ab)	3.834	0.010

One-way analysis of variance (ANOVA) was used for group comparisons. Letters in parentheses indicate post hoc pairwise comparisons; values sharing the same letter did not differ significantly. Statistical significance was defined as *p* < 0.05.

**Table 4:** Distribution of Braden Risk Categories According to Interventional Procedures among Patients with Documented Pressure Injuries.

Variable		Braden Risk Category								χ^2^-Value	*p*-Value
		12 and Below, High Risk		13–14, Moderate Risk		15–16, Low Risk		17 and Upper, No Risk			
		*n*	%	*n*	%	*n*	%	*n*	%		
Foley Catheter	Absent	14	38.9 (a)	10	27.8	9	25.0 (a)	3	8.3	15.096	0.002
	Present	162	66.7 (b)	47	19.3	18	7.4 (b)	16	6.6		
NG, Oxygen Mask, Nasal Cannula	Absent	61	53.0 (a)	30	26.1 (a)	14	12.2	10	8.7	8.471	0.037
	Present	115	70.1 (b)	27	16.5 (b)	13	7.9	9	5.5		

Pearson’s chi-square test was used. Letters in parentheses indicate pairwise comparisons between categories; values sharing the same letter are not significantly different. Statistical significance was set at *p* < 0.05. NG = nasogastric tube.

**Table 5:** Distribution of Braden Risk Categories According to Pressure Injury Status at Admission or During Hospitalization.

Variable		Braden Risk Category								χ^2^-Value	*p*-Value
		12 and Below, High Risk		13–14, Moderate Risk		15–16, Low Risk		17 and Upper, No Risk			
		*n*	%	*n*	%	*n*	%	*n*	%		
Pressure injury status	Yes	139	64.1	47	21.7	20	9.2	11	5.1	5.428	0.143
	Developed during hospitalization	37	59.7	10	16.1	7	11.3	8	12.9		

Pearson’s chi-square test was used. Statistical significance was set at *p* < 0.05.

**Table 6:** Distribution of Pressure Injury Sites and Stages According to Primary Diagnosis.

Variable		Diagnosis												χ^2^-Value	*p*-Value
		Oncological		Acute Traumas		Neurological		Cardiology		Internal Medicine		Other			
		*n*	%	*n*	%	*n*	%	*n*	%	*n*	%	*n*	%		
Sacrum	0	31	17.7	3	5.0	6	18.2	2	40.0	0	0.0	0	0.0	64.065	<0.001
	1	63	36.0 (a)	13	21.7 (a)	4	12.1 (a)	1	20.0 (a)	2	33.3 (a)	0	0.0		
	2	63	36.0 (a)	17	28.3 (a)	11	33.3 (a)	1	20.0 (a)	2	33.3 (a)	0	0.0		
	3	13	7.4 (a)	9	15.0 (a)	6	18.2 (a)	1	20.0 (a)	0	0.0 (a)	0	0.0		
	4	3	1.7 (a)	8	13.3 (b)	4	12.1 (b)	0	0.0 (ab)	0	0.0 (ab)	0	0.0		
	5	2	1.1 (a)	10	16.7 (b)	2	6.1 (ab)	0	0.0 (ab)	2	33.3 (b)	0	0.0		
Trochanter	0	152	86.9	42	70.0	24	72.7	4	80.0	5	83.3	0	0.0	47.443	0.001
	1	10	5.7 (a)	2	3.3 (a)	1	3.0 (a)	0	0.0 (a)	0	0.0 (a)	0	0.0		
	2	11	6.3 (a)	6	10.0 (a)	2	6.1 (a)	0	0.0 (a)	0	0.0 (a)	0	0.0		
	3	1	0.6 (a)	6	10.0 (b)	2	6.1 (ab)	1	20.0 (b)	0	0.0 (ab)	0	0.0		
	4	0	0.0 (a)	1	1.7 (ab)	3	9.1 (b)	0	0.0 (ab)	0	0.0 (ab)	0	0.0		
	5	1	0.6 (a)	3	5.0 (ab)	1	3.0 (ab)	0	0.0 (ab)	1	16.7 (b)	0	0.0		
Heel	0	157	89.7	41	68.3	21	63.6	3	60.0	4	66.7	0	0.0	54.886	<0.001
	1	6	3.4 (a)	3	5.0 (a)	4	12.1 (a)	0	0.0 (a)	1	16.7 (a)	0	0.0		
	2	9	5.1 (a)	10	16.7 (b)	3	9.1 (ab)	0	0.0 (ab)	0	0.0 (ab)	0	0.0		
	3	1	0.6 (a)	4	6.7 (b)	2	6.1 (ab)	2	40.0 (b)	1	16.7 (b)	0	0.0		
	4	1	0.6 (a)	1	1.7 (a)	1	3.0 (a)	0	0.0 (a)	0	0.0 (a)	0	0.0		
	5	1	0.6 (a)	1	1.7 (a)	2	6.1 (a)	0	0.0 (a)	0	0.0 (a)	0	0.0		

Pearson’s chi-square test was used. Each anatomical pressure injury site was evaluated separately. Because some patients had pressure injuries at more than one anatomical site, percentages across anatomical sites may not sum to 100%. Letters in parentheses indicate pairwise comparisons between diagnostic categories; values sharing the same letter are not significantly different. Statistical significance was set at *p* < 0.05. Stage coding: 0 = no pressure injury at the specified anatomical site; 1 = Stage I; 2 = Stage II; 3 = Stage III; 4 = Stage IV; 5 = unstageable/deep tissue pressure injury.

**Table 7:** Pressure Injury Status at Admission or During Hospitalization According to Primary Diagnosis.

Variable		Diagnosis												χ^2^-Value	*p*-Value
		Oncological		Acute Traumas		Neurological		Cardiology		Internal Medicine		Other			
		*n*	%	*n*	%	*n*	%	*n*	%	*n*	%	*n*	%		
Pressure injury status	Yes	122	69.7 (a)	56	93.3 (b)	30	90.9 (ab)	4	80.0 (ab)	5	83.3 (ab)	0	0.0	18.397	0.001
	Developed during hospitalization	53	30.3 (a)	4	6.7 (b)	3	9.1 (ab)	1	20.0 (ab)	1	16.7 (ab)	0	0.0		

Pearson’s chi-square test was used. Letters in parentheses indicate pairwise comparisons between categories; values sharing the same letter are not significantly different. Statistical significance was set at *p* < 0.05.

**Table 8:** Pressure Injury Status at Admission or During Hospitalization According to Caregiver Degree of Kinship.

Variable		Caregiver Degree of Kinship						χ^2^-Value	*p*-Value
		1. Degree		2. Degree		Care Worker			
		*n*	%	*n*	%	*n*	%		
Pressure injury status	Yes	190	87.6 (a)	14	6.5 (a)	13	6.0 (a)	7.249	0.027
	Developed during hospitalization	51	82.3 (a)	10	16.1 (b)	1	1.6 (a)		

Pearson’s chi-square test was used. Letters in parentheses indicate pairwise comparisons between categories; values sharing the same letter are not significantly different. Statistical significance was set at *p* < 0.05.

**Table 9:** Pressure Injury Status at Admission or During Hospitalization According to Level of Consciousness.

Variable		State of Consciousness						χ^2^-Value	*p*-Value
		Conscious		Confused		Unconscious			
		*n*	%	*n*	%	*n*	%		
Pressure injury status	Yes	80	72.1 (a)	93	76.9 (a)	44	93.6 (b)	8.972	0.011
	Developed during hospitalization	31	27.9 (a)	28	23.1 (a)	3	6.4 (b)		

Pearson’s chi-square test was used. Letters in parentheses indicate pairwise comparisons between categories; values sharing the same letter are not significantly different. Statistical significance was set at *p* < 0.05.

**Table 10:** Pressure Injury Status at Admission or During Hospitalization According to Mobility Status.

Variable		Mobility Status								χ^2^-Value	*p*-Value
		Mobile		Mobile with Assistance		Mobile in Bed		Immobil			
		*n*	%	*n*	%	*n*	%	*n*	%		
Pressure injury status	Yes	2	100.0 (ab)	29	59.2 (b)	35	67.3 (b)	151	85.8 (a)	20.217	<0.001
	Developed during hospitalization	0	0.0 (ab)	20	40.8 (b)	17	32.7 (b)	25	14.2 (a)		

Pearson’s chi-square test was used. Letters in parentheses indicate pairwise comparisons between categories; values sharing the same letter are not significantly different. Statistical significance was set at *p* < 0.05.

**Table 11:** Multivariable Binary Logistic Regression Analysis of Factors Associated with Pressure Injury Development During Hospitalization among Patients with Documented Pressure Injuries.

Variable	Category/Comparison	Adjusted OR	95% CI	*p*-Value
Primary diagnosis	Acute trauma vs. oncology	0.21	0.07–0.67	0.008
Primary diagnosis	Neurological vs. oncology	0.33	0.09–1.23	0.099
Primary diagnosis	Other diagnoses vs. oncology	0.49	0.09–2.52	0.391
Caregiver degree of kinship	Second-degree relative vs. first-degree relative	2.93	1.12–7.71	0.029
Caregiver degree of kinship	Care worker vs. first-degree relative	0.55	0.07–4.65	0.586
Level of consciousness	Confused vs. conscious	1.52	0.74–3.12	0.258
Level of consciousness	Unconscious vs. conscious	0.34	0.08–1.41	0.138
Length of stay	Per one-day increase	1.02	1.01–1.04	0.005
Mobility status	Immobile vs. non-immobile	0.44	0.21–0.91	0.027

Note. Binary logistic regression analysis was performed. The dependent variable was pressure injury status, OR = odds ratio; CI = confidence interval. Statistical significance was set at *p* < 0.05.

## Data Availability

The datasets are available from the correspondent authors upon reasonable request due to local policies.
